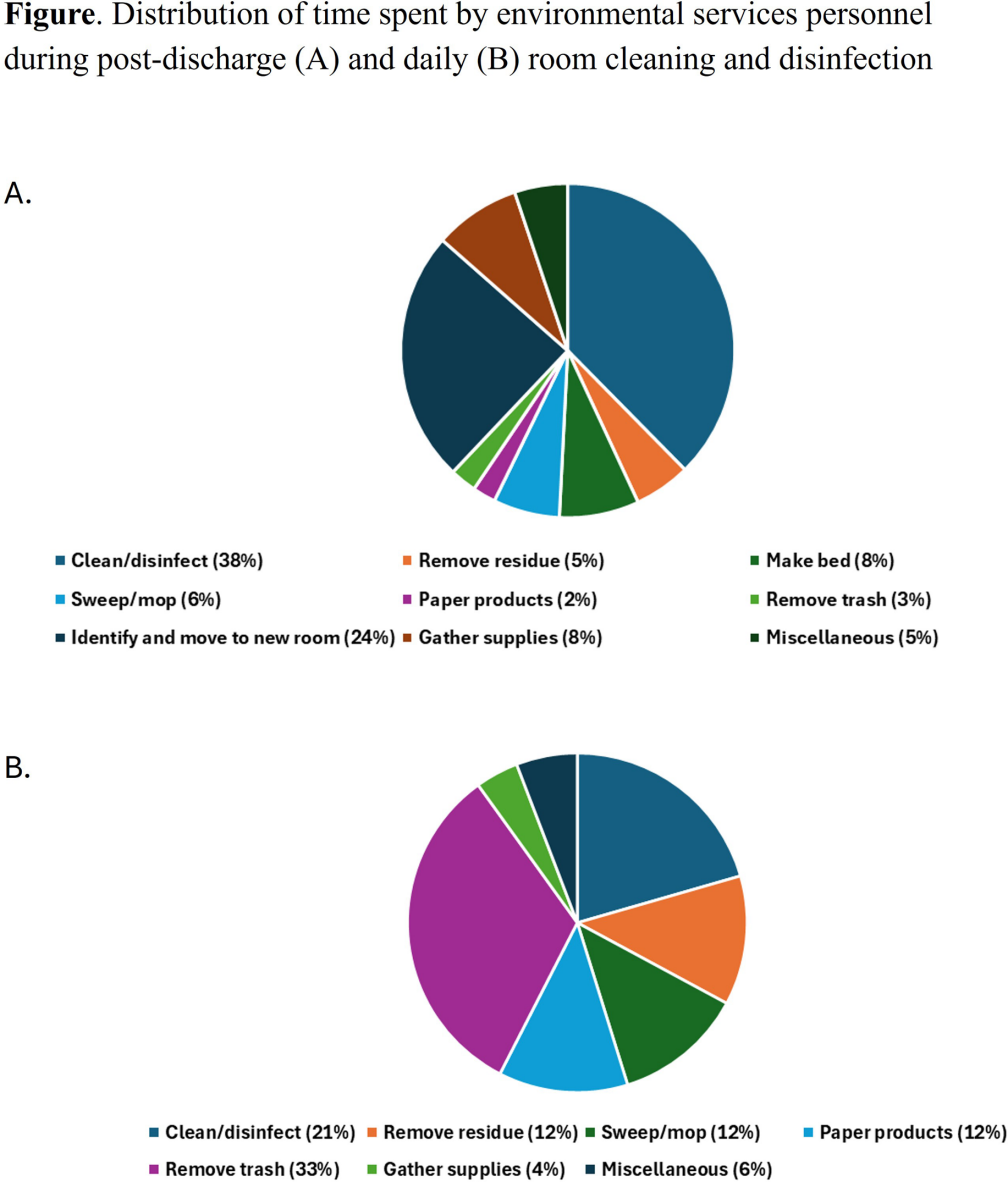# 224 Decolonization Dilemmas: What’s Current and What’s Needed—A Survey of the SHEA Research Network and Affiliated US-based Hospitals

**DOI:** 10.1017/ash.2026.10604

**Published:** 2026-06-23

**Authors:** Jennifer Cadnum, Amelia Milner, Samir Memic, Curtis Donskey

**Affiliations:** 1 Cleveland VA Medical Center; 2 VA Medical Center

## Abstract

**Background:** Environmental services (EVS) programs may choose dilutable (i.e., concentrate diluted manually or with an automated dispensing system) or ready-to-use (RTU) disinfectant products. Dilutable products are less expensive but have greater potential for incorrect use. **Methods:** We conducted a time and motion evaluation of EVS cleaning and disinfection practices before and after a facility-wide switch from dilutable to RTU quaternary ammonium disinfectant products. Observations were completed to determine the time required to complete tasks and the appropriateness of product use (e.g., correct product, contact time). Personnel were graded using a standardized compliance scale (17-20, highly compliant; 14-16 moderately compliant; 10-13, needs improvement). **Results:** We conducted 40 total hours of observations of 8 EVS personnel before and 10 after the product substitution. Mean compliance scores increased from 14.3 to 18.2 after the substitution. Noncompliance when dilutable products were used was most often due to incorrect use of products, including inappropriate mixing of disinfectant products (e.g., dilutable quaternary ammonium plus sodium hypochlorite) and inadequate contact time. During post-discharge and daily cleaning before the substitution, activities other than cleaning and disinfection accounted for 50% and 55% of total EVS personnel time, respectively (Figure), with similar time distribution after the substitution. **Conclusions:** Switching from dilutable to RTU disinfectants improved compliance with recommended cleaning and disinfection practices. EVS personnel spend a substantial proportion of their time doing activities other than cleaning and disinfection.

**Figure:**